# First Experience with Extracorporeal Membrane Oxygenation in Iran, under Difficult Conditions

**Published:** 2018-10

**Authors:** Zargham Hossein Ahmadi, Farshid Salehi, Shanay Niusha, Mohammad Reza Raoufy, Behrooz Farzanegan, Ali Afshar, Yadollah Mafhoomi, Zahra Faghih Abdollahi, Golnar Radmand, Zahra Ansari Aval, Alireza Jahangirifard

**Affiliations:** 1 *Lung Transplantation Research Center, National Research Institute of Tuberculosis and Lung Diseases (NRITLD), Shahid Beheshti University of Medical Sciences, Tehran, Iran.*; 2 *Tracheal Diseases Research Center, National Research Institute of Tuberculosis and Lung Diseases (NRITLD), Shahid Beheshti University of Medical Sciences, Tehran, Iran.*; 3 *Department of Physiology, Faculty of Medical Sciences, Tarbiat Modares University, Tehran, Iran.*; 4 *Nursing and Respiratory Health Management Research Center, National Research Institute of Tuberculosis and Lung Diseases (NRITLD), Shahid Beheshti University of Medical Sciences, Tehran, Iran.*; 5 *Anesthesiology Research Center, * *Shahid Beheshti University of Medical Sciences, Tehran, Iran.*; 6 *Pediatric Respiratory Diseases Research Center, National Research Institute of Tuberculosis and Lung Diseases (NRITLD), Shahid Beheshti University of Medical Sciences, Tehran, Iran.*

**Keywords:** *Extracorporeal membrane oxygenation*, *Heart failure*, *Respiratory insufficiency*

## Abstract

**Background:** Extracorporeal membrane oxygenation (ECMO) provides hemodynamic and oxygenation support in critical conditions. The commencement of this modality in Iran coincided with severe economic constraints across Iran.

**Methods:** This retrospective study was performed in Masih Daneshvari Medical Center from 2010 to 2015, during which period, sanction-related limitations in the import of equipment prompted us to integrate a Medtronic or Stöckert head pump console into a Maquet ECMO Oxygenator so as to sustain the ECMO program. Comparisons were performed between successful and unsuccessful ECMO procedures and survivors. Factors associated with unsuccessful ECMO were evaluated with a multivariate logistic regression.

**Results:** Thirty-three (68.8%) patients were male and 15 (31.2%) were female. The mean age of the patients was 35±16.6 years. Thirty-seven (77.1%) patients were weaned off ECMO successfully; the rate was higher than that in previous studies. Totally, 35.4% of the study population survived to hospital discharge. The most common cause of death in all the ECMO patients who were successfully weaned was sepsis. The most common cause of death in the patients who underwent unsuccessful ECMO was multisystem organ failure. The mean ECMO support time was 53.37±46.26 hours. The patients who were alive at discharge were significantly younger (25.5±14.5 vs. 40.2±15.5 y; P=0.002) and had a significantly lower ECMO duration (24 [25–75% interquartile: 18.5–36] vs. 48 [25–75% interquartile: 24–72] h; P=0.044) than the non-survivors.

**Conclusion:** An assembly of ECMO components from different companies could be done safely, at least for a short period of time.

## Introduction

Extracorporeal membrane oxygenation (ECMO), a modification of partial cardiopulmonary bypass (CPB), is an extracorporeal technique to provide support for the respiratory or cardiac function. It works by removing the blood from the body, artificially extracting its carbon dioxide, oxygenating its red blood cells, and then returning it to the body, either peripherally or centrally. In other words, it is a prolonged type of CPB.

ECMO was introduced in 1972, since which time it has gradually become an accepted mode of treatment not only in a wide range of age,^1^ from neonates to adults, but also in various reversible heart and lung diseases and irreversible heart and lung diseases as a bridge to transplantation^2^ as well as in septic shock,^3^ H1N1,^4^ adult respiratory distress syndrome (ARDS),^5^ and after cardiopulmonary resuscitation.^6^

The main components of the ECMO circuit are the oxygenator and the pump. When being drained from the outflow cannula, blood enters the pump and is driven through the oxygenator. The oxygenated blood is returned to the patient via the inflow cannula.^7^ Oxygenators, biopumps, cannulae, and tubes are removable segments, and they should be replaced for each patient. 

Whereas ECMO has been a staple of the routine management of temporary circulatory or respiratory failure in Western countries, it has not been applicable in our country due to its high cost. In Iran, the ECMO program was started in 2010 in Masih Daneshvari Medical Center, National Research Institute of Tuberculosis and Lung Disease (NRITLD), Shahid Beheshti University of Medical Sciences, Tehran. This hospital is a tertiary center with large numbers of patients with respiratory failure and is also a part of an active heart and lung transplantation program. The centrifugal head pump console used was Medtronic and Stöckert. Between 2010 and 2015, economic sanctions imposed on Iran owing to its nuclear energy programs made the acquisition of the ECMO oxygenator sets of the aforementioned companies impossible, and the only available ECMO oxygenator was Maquet (PLS set). Therefore, a Medtronic or Stöckert head pump was integrated with a Maquet ECMO oxygenator in order to continue the ECMO program.^4^

Previous studies have reported the survival outcomes after ECMO support. The survival rate of ECMO in neonates with respiratory failure has been reported to be approximately 90%; nonetheless, in adults with respiratory failure, it has gradually progressed from 9% to between 39% and 54%.^8^

The aim of the present study was to describe our experience as a single ECMO center during a period of time when due to limitations we were forced to assemble the different parts of ECMO from different companies in order to save our patients. This practice is not recommended by the companies. 

No study has evaluated the survival rates of ECMO in circumstances where the oxygenator and the console pump came from 2 different companies and, thus, did not completely match. Herein, we present our institutional experience with respect to the survival and mortality rates of an ECMO circuit assembled with unmatched oxygenators and centrifugal pumps from 2010 to 2015. This is also the first experience of ECMO in Iran. This study was approved and closely observed by the Ethics Committee of the National Research Institute of Tuberculosis and Lung disease (NRITLD). 

## Methods

Between July 23, 2010 and June 6, 2015, 48 patients underwent ECMO in our center.

The indication of operation depended on each patient’s condition. For patients with intractable heart failure, ECMO was used in the presence of a maximum persistent systolic blood pressure of 80 mm Hg and a decreased urine output of less than 0.5 cc/kg/min, even if high doses of inotropes were used. 

ECMO was utilized for patients with respiratory problems when the ratio of arterial oxygen partial pressure to fractional inspired oxygen (PaO_2_/FiO_2_) was less than 100 while the patient was ventilated with 100% oxygen and maximal ventilatory support.

The exclusion criteria were according to the guidelines of the Extracorporeal Life Support Organization (ELSO) and comprised age older than 65 years, obesity, multisystem organ failure, irreversible and/or significant neurological impairment, intracranial bleeding, coagulopathy, prolonged mechanical support more than 10 days, and weight over 140 kg. 

From 2010 to 2011, a conventional centrifugal pump (550 Bio-Console^®^ Medtronic BioMedicus^®^, Minneapolis, MN, USA) was used. At the time, the only available hollow fiber oxygenator in our country was the ECMO oxygenator of the Maquet PLS Set (Maquet Getinge Group, Rastatt Germany), the biopump of which was designed for use by Jostra Head Pumps. Therefore, the head pump of this set was replaced with a Medtronic Biopump BPX-80 (Medtronic^®^, Minneapolis, MN, USA). In 2011, this set was replaced with the Stöckert Centrifugal Pump Console (Sorin Group Deutschland GmbH Lindberghstraße, München, Germany) and the EO Sorin Oxygenator (Mirandola, Modena, Italy). After a few months, it was not possible to import Sorin oxygenators due to additional limitations in our country. Meanwhile, the only available oxygenator was still the Maquet ECMO Oxygenator (PLS Set, Maquet Getinge Group, Rastatt Germany), although the high costs made it impossible to supply the Maquet Console for our hospital. Therefore, in order to continue the ECMO program, we had no choice but to combine or assemble a Medtronic or Stöckert head pump with a Maquet ECMO Oxygenator. Both femoral arterial and venous cannulae (19–21 F) (Medtronic^®^, Minneapolis, MN, USA) were used with our ECMO set.

Veno-venous (VV) ECMO was established for pure respiratory failure without any evidence of concomitant alteration in the cardiac function, which did not respond to mechanical ventilation. For other patients with cardiac or combined cardiac and respiratory support, such as ARDS patients with a low blood pressure, veno-arterial (VA) ECMO was established.

The ECMO blood flow was adequately adjusted in the first 24 to 48 hours to provide a maximal flow (60 cc/kg/min), and then it was gradually decreased to obtain sufficient support. Since mixed venous oxygen saturation is the best criterion for the evaluation of patients, efforts were made to maintain it around 70% at the beginning. Usually, at the beginning of ECMO, high doses of inotropes were used before they were gradually reduced according to the perfusion pressure to maintain a mean arterial pressure of 50 to 70 mm Hg. In the first 24 hours, the patients were kept deeply sedated and even paralyzed and after that period, the dose of the sedatives and the paralytic agents was decreased if the patients could tolerate ECMO support. Continuous intravenous heparin was administered as an anticoagulant to achieve an activated clotting time of 160 to 200 seconds. ECMO support was conducted under normothermia at 36^°^C to 37^°^C by applying a heat exchanger (Medtronic^®^, Minneapolis, MN, USA) and a blanket. Platelet count was kept up above 80 000 and hematocrit was kept above 30% in VA ECMO and 35% in VV ECMO. The venous drainage negative pressure was maintained within 30 mm Hg. The oxygenator membrane absorbed oxygen at a concentration of 60%. Mechanical ventilation was continued throughout ECMO support, with its setting mode differing according to the lung capacity of each patient. The aim was to keep PaO_2_ between 60 and 150 mm Hg in VA ECMO and between 45 and 80 mm Hg in VV ECMO. For the patients with a high airway pressure and a poor lung compliance, pressure controlled ventilation was applied. For the other patients, synchronized intermittent mandatory ventilation and pressure support were used. The ventilator setting was commonly set at a tidal volume of 6 to 8 mL/kg with a pressure support of 10 cm H_2_O, 8 to 10 breaths/min, positive end-expiratory pressure of 5 to 15 mm Hg, FiO_2_ of 30 to 50% with a peak airway pressure of 25 to 30 cm H_2_O, and inspiratory/expiratory rate of 2/1 to 1/1. Tracheostomy was performed on all the patients after 5 days.

Weaning was considered when the function of the heart or lung or both returned to normal or near normal and less than 30% of ECMO support could be tolerated by the patient. Accordingly, the parameter settings of the ventilator and the doses of the inotropes were adjusted. The ECMO flow was gradually reduced according to the perfusion pressure and the urine output. If the ECMO flow of 1000 mL/min was tolerated by the patient for 1 to 2 hours, the device was removed. Therefore, the ECMO cannula was removed in the operating room or in the intensive care unit (ICU).

Weaning with regard to VA ECMO consisted of decreasing the flow of the drainage and the infusion lines and adjusting the doses of the inotropes and the setup of the ventilator. Once the arterial blood gas and the patient’s hemodynamics remained stable after 1 to 2 hours, ECMO was explanted.

Concerning VV ECMO, first the ECMO flow was reduced to 1000 mL/min and the gas flow was stopped, and then the ventilator setting was altered to post-weaning parameters. If no significant changes occurred in the arterial oxygen saturation and in the partial pressure of arterial carbon dioxide within 1 hour and the patient was stable, then the ECMO circuit could be removed.

Chest radiography and echocardiography were done daily to assess the heart and lung functions. Blood profile, biochemical markers, and arterial blood gases were routinely examined at least 4 times daily.

Weaning off the ventilator after weaning off ECMO support was done according to the standard protocols.

The data are presented as means±standard deviations (SDs) for the normally distributed variables, as medians and 25th and 75th percentiles for those with non-normal distributions, and as numbers and percentages for the categorical variables. The significance of the differences between the groups was assessed with a *t*-test for age, a Mann–Whitney test for the duration of ECMO, and a Fisher exact test for all the other variables. Receiver operating characteristic (ROC) curves were utilized to determine the diagnostic powers and the best cutoff points of age and the ECMO duration for the prediction of being alive at discharge. A logistic regression analysis was applied to evaluate the predictive value of age and the ECMO duration, with being alive at discharge regarded as the outcome. A P value of less than 0.05 was considered significant. The calculations were done with GraphPad Prism, version 3.0 (GraphPad Software, San Diego, CA), and the SPSS package, version 15 (SPSS, 1989-2006, Chicago, Illinois, USA).

## Results

Between June 2010 and July 2015, a total of 48 patients underwent the ECMO procedure in our center. The mean age of the patients was 35.0±16.6 years. Thirty-three (68.8%) patients were male and 15 (31.2%) were female.

The indications for ECMO support were H1N1 (n=2), AIDS (n=1), heart failure (3 patients), post heart transplantation (n=3), post lung transplantation (n=24), lung lavage (n=5), pulmonary embolectomy (n=1), pulmonary endarterectomy (n=3), ARDS (n=6), and carinal tumor (n=1).

Thirty-seven (77.1%) patients were weaned off ECMO successfully. Among the patients who were weaned off ECMO (n=37), 17 patients (45.9% of the successful ECMO procedures and 35.4% of the total study population) survived to hospital discharge. In other words, as is depicted in Table 1, the patients who were alive at discharge were significantly younger (25.5±14.5 vs. 40.2±15.5 y; P=0.002) and had significantly lower ECMO durations (24 [25–75% interquartile: 18.5–36] vs. 48 [25–75% interquartile: 24–72) h; P=0.044) than the non-survivors (Table 1). 

The rate of successful weaning and being alive at discharge in the heart-failure group was 83.3% (5 out of 6 patients) and 33.3% (2 out of 6 patients), respectively, while in the lung-failure group, this rate was 81.1% (30 out of 37 patients) and 40.5% (15 out of 37 patients).

As is shown in Table 2, there was a significant difference in the reason for ECMO as well as in the cause of death between the patients with successful and unsuccessful weaning (P=0.025 and P=0.006, respectively). Lung transplantation (56.8%) and ARDS (36.4%) were the most common reasons for ECMO in the patients with successful and unsuccessful weaning, correspondingly. Sepsis (70%) and multisystem organ failure (63.3%) were the most common causes of death in the patients with successful and unsuccessful weaning, respectively.

The most common cause of death in the unsuccessful ECMO procedures was bleeding, and the most common cause of death in the successful ECMO procedures was sepsis (P=0.006).

**Figure 1:**
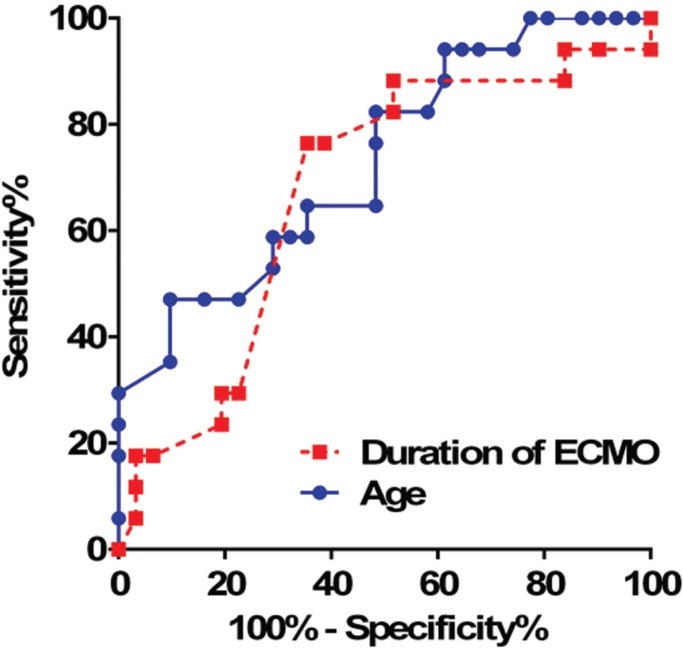
Receiver operating characteristic (ROC) curves for age and the duration of extracorporeal membrane oxygenation (ECMO) to predict being alive at discharge

ROC curves were used to determine the diagnostic powers and the best cutoff points of age and the ECMO duration for the prediction of being alive at discharge (Figure1). Both age and the ECMO duration had good diagnostic powers for the prediction of being alive at discharge (age: AUC=0.732, 95%CI: 0.417–0.772, P=0.009; and ECMO duration: AUC=0.675, 95%CI: 0.513–0.836, P=0.047). The best cutoff point of age for the prediction of being alive at discharge was less than 29 hours, yielding a sensitivity of 82.4% (95%CI: 56.6–96.2) and a specificity of 51.6% (95%CI: 33.1–69.9). The best cutoff point of the ECMO duration was less than 35.5 years, yielding a sensitivity of 76.5% (95%CI: 50.1–93.2) and a specificity of 64.5% (95%CI: 45.4–80.8).

A logistic regression analysis was used to evaluate the predictive value of age (<35.5 y) and the ECMO duration (<29 h), with being alive at discharge regarded as the outcome. As is illustrated in Table 3, the ECMO duration was a significant predictor of being alive at discharge in the univariate logistic regression analysis (OR=0.169, P=0.009) and in the multivariate logistic regression analysis (OR=0.193, P=0.021). However, age was a predictor of being alive at discharge in the univariate logistic regression analysis (OR=0.201, P=0.028) but not in the multivariate logistic regression analysis (OR= 0.237, P=0.062).

**Table 1:** Characteristics of the patients who are alive the discharge*

	Total	Dead (n=31)	Alive at Discharge (n=17)	P
Age (y)	35.03±16.61	40.21±15.53	25.54±14.53	0.002
Male	33 (68.8)	21 (67.7)	12 (70.6)	0.999
Duration of ECMO (hr)	29 (22-72)	48 (24-72)	24 (18.5-36)	0.044
Cause of ECMO				0.081
AIDS	1 (2.1)	1 (3.2)	0	
H1N1 influenza	2 (4.2)	1 (3.2)	1 (5.9)	
Post cardiotomy	3 (6.3)	3 (9.7)	0	
Heart transplantation	3 (6.3)	2 (6.5)	1 (5.9)	
Alveolar proteinosis	4 (8.3)	0	4 (23.5)	
Lung transplantation	24 (50.0)	15 (48.4)	9 (52.9)	
Pulmonary embolectomy	1 (2.1)	1 (3.2)	0	
Pulmonary endarterectomy	3 (6.3)	2 (6.5)	1 (5.9)	
ARDS	6 (12.5)	6 (19.4)	0	
Carinal tumor	1 (2.1)	0	1 (5.9)	
Failed organs				0.204
Heart failure	6 (12.5)	4 (12.9)	2 (11.8)	
Lung failure	37 (77.1)	22 (71.0)	15 (88.2)	
Sepsis	5 (10.4)	5 (16.1)	0	

The significance of the differences between the groups was assessed with a *t*-test for age, a Mann–Whitney test for the duration of ECMO, and a Fisher exact test for all the other variables.

ECMO, Extracorporeal membrane oxygenation; AIDS, Acquired immune deficiency syndrome; ARDS, Adult respiratory distress syndrome

* Data are presented as mean±SD for age, as median (25–75%) for the duration of ECMO, and as number (%) for all the other variables.

**Table 2:** Characteristics of the patients with successful ECMO*

	Total	Unsuccessful (n=11)	Successful (n=37)	P
Age (y)	35.03±16.61	39.61±15.94	33.64±16.82	0.303
Male	33 (68.8)	7 (63.6)	26 (70.3)	0.720
Duration of ECMO (hr)	29 (22-72)	24 (22-72)	34 (21-72)	0.812
Alive at discharge	17 (35.4)	0	17 (45.9)	0.004
Cause of ECMO				0.025
AIDS	1 (2.1)	1 (9.1)	0	
H1N1 influenza	2 (4.2)	1 (9.1)	1 (2.7)	
Post cardiotomy	3 (6.3)	0	3 (8.1)	
Heart transplantation	3 (6.3)	1 (9.1)	2 (5.4)	
Alveolar proteinosis	4 (8.3)	0	4 (10.8)	
Lung transplantation	24 (50.0)	3 (27.3)	21 (56.8)	
Pulmonary embolectomy	1 (2.1)	1 (9.1)	0	
Pulmonary endarterectomy	3 (6.3)	0	3 (8.1)	
ARDS	6 (12.5)	4 (36.4)	2 (5.4)	
Carinal tumor	1 (2.1)	0	1 (2.7)	
Failed organs				0.113
Heart failure	6 (12.5)	1 (9.1)	5 (13.5)	
Lung failure	37 (77.1)	7 (63.6)	30 (81.1)	
Sepsis (Heart and Lung failure)	5 (10.4)	3 (27.3)	2 (5.4)	
Cause of death				0.006
Asphyxia	1 (3.2)	1 (9.1)	0	
Bleeding	4 (12.9)	1 (9.1)	3 (15.0)	
CVA	1 (3.2)	0	1 (5.0)	
MSOF	8 (25.8)	7 (63.6)	1 (5.0)	
Rejection	1 (3.2)	0	1 (5.0)	
Sepsis	16 (51.6)	2 (18.2)	14 (70.0)	

The significance of the differences between the groups was assessed with a *t*-test for age, a Mann–Whitney test for the duration of ECMO, and a Fisher exact test for all the other variables.

ECMO, Extracorporeal membrane oxygenation; ARDS, Adult respiratory distress syndrome; CVA, Cerebrovascular accidents; MSOF, Multisystem organ failure

* Data are presented as mean±SD for age, as median (25–75%) for the duration of ECMO, and as number (%) for all the other variables.

**Table 3:** Predictors of being alive at discharge, as determined by logistic regression

	Univariate Analysis		Multivariate Analysis	
	OR (95%CI)	P	OR (95%CI)	P
Age: <35.5 vs. ≥35.5 y	0.201 (0.048-0.841)	0.028	0.237 (0.052-1.075)	0.062
Duration of ECMO: <29 vs. ≥29 hr	0.169 (0.044-0.647)	0.009	0.193 (0.048-0.779)	0.021

ECMO, Extracorporeal membrane oxygenation

## Discussion

ECMO appears to have recently revolutionized the management of life-threatening pulmonary or cardiac failure when other forms of treatment have proved somewhat unlikely to succeed. ECMO was introduced in 1970s,^1^ and the recent years have witnessed its progressive growth on the strength of its undeniable advantages and role in increasing the survival rate of patients. Indeed, ECMO’s cheap costs relative to those of other more complex mechanical supports,^9^ easy implantability through peripheral or central vessels, feasibility to install in or outside the operating room, and swiftness as a procedure are examples of advantages that have made ECMO an accepted mode of treatment.^10^

For all these advantages of ECMO and its indisputable role in critically ill patients, however, the complication and mortality rates in patients who undergo ECMO are still high. The reported survival rates of ECMO support vary in different studies, from 28 to 63%. ^10^^- ^^14^ The disparity in the results is mainly in consequence of the different patient-selection protocols and the different reasons for the utilization of ECMO. The worst outcome was in our post-cardiotomy patients, who could not be weaned off CPB. Similar results have also been reported by other studies.^12^ In a meta-analysis by Zangrillo et al.,^15^ the data of 12 studies were summarized. The overall mortality rate in this meta-analysis was 54% (which also showed 55% successful weaning). The ELSO, an international consortium of health-care institutions established in 1989, maintains a registry of all known cases in which ECMO is used.^16^^, ^^17^ In the adult population (>18 y), 47.5% of the patients who underwent ECMO support survived to discharge from the hospital, while 58.6% of all the patients were weaned from ECMO successfully. A comparison of these data with our findings showed that our successful weaning rate (77.1%) was higher than that of the previous studies. Our higher rate of successful weaning suggests that, although our ECMO circuit was a combination of unmatched oxygenators and head pumps, the function of our ECMO device was acceptable and the patients were weaned without any organ dysfunction.

In previous studies, some patients treated with ECMO for a respiratory indication had better outcomes than the patients who had a cardiac indication and they were more likely to have a better 1-year survival rate.^2^^, ^^8^^, ^^18^ In contrast, we found no difference between the heart, lung, and heart and lung failure groups. This may be due to a short ECMO duration for our patients by comparison with that reported by the previous studies. The mean duration of ECMO in our study was 2 days (maximum: 7.5 d and minimum: 10 h). The least duration of ECMO was in the lung transplantation patients whose vital signs and oxygenation became normal after transfer to the ICU and a decision was made to wean them off after 10 hours. 

However, compared with the previous studies,^11^^, ^^13^^, ^^18^ in our study, the percentage of the patients who were weaned off ECMO and who survived to discharge alive was low (35.4%). The most common cause of death in our patients who underwent unsuccessful ECMO was multisystem organ failure (70%), followed by sepsis (20%) and bleeding (10%). These results are consistent with those of some other investigations.^10^^, ^^11^ Nevertheless, unlike the previous studies,^10^^, ^^13^^, ^^15^ in our center, the most common cause of death in the successfully weaned patients was sepsis (51.6%). In the literature, the incidence of the occurrence of infections during ECMO ranges widely from 3.5% to 45.1%,^19^^,^^20^ with bloodstream infection constituting the most common infection insofar as it occurs in 32.6% to 89.4% of these infections.^20^^-^^22^ It appears that a longer duration of ECMO, some mechanical complications, immune system suppression or the use of steroids for underlying diseases, and the placement of multiple central catheters can lead to an increased risk of infection and sepsis.^19^^, ^^21^^,^^22^ Meanwhile, the duration of ECMO in our patients was not too long, but the setting of our ICU was not as sophisticated as the ICUs in Western countries and isolation was not restricted. The contaminations may have occurred during assembly and initiated bacteremia and subsequent sepsis, which occurred in all the patients, especially the heart transplantation and lung transplantation patients who received immunosuppressants. Bleeding is one of the most common and serious complications of ECMO insofar as it is seen in about 8% to 20% of the cases.^3^^, ^^10^^,^^18^ Bleeding was the cause of death in 1 (9.1%) patient among all the unsuccessful ECMO patients. Multisystem organ failure was also seen in 7 (63.6%) of our patients, which was mostly due to a low flow state and the late application of ECMO. This complication was the major cause of mortality in the other studies.^11^^, ^^19^ In the previous investigations, cerebrovascular accidents and ischemic complications accounted for 4% to 9% of the mortality rates.^5^^, ^^18^ One (3.2%) of our patients suffered a hemorrhagic cerebrovascular accident. 

Apropos organ failure, it has been suggested that sepsis be classified as a case of both heart and lung failure. It is deserving of note that in the case of heart failure, we established VA ECMO, in the case of lung failure VV ECMO, and in the case of both heart and lung failure VA ECMO or VAV ECMO. Although the best results were obtained in the heart-failure group and the worst result in the sepsis group, we found no statistically significant difference between these groups (P=0.113).

The predictors of being alive at discharge were age younger than 35 years and a duration of ECMO of less than 29 hours. The duration may seem very short, but it should be noted that we used medians in lieu of means because of the non-normal distribution of the duration of ECMO. 

In all these cases, the ECMO oxygenator used was the Maquet PLS Set and all the circuits were Bioline-coated, while the cannulae were from Medtronic Company. The main change was replacing the Stöckert or Medtronic centrifugal pump with the Rota flow drive of the Maquet Oxygenator. During ECMO, there was no plasma leakage or any malfunctioning of the oxygenator, and nor was there any evidence of renal failure or jaundice in favor of massive hemolysis, although the mean duration of ECMO was not prolonged. 

A low sample size and the retrospective design of the current study are its most salient drawbacks. In addition, there was no controlled study with which to compare the results of our study, and a lack of facilities precluded the measurement of free hemoglobin levels to evaluate the possibility of hemolysis. 

## Conclusion

A successful ECMO procedure requires high expenses and well-structured teams of highly skilled staff. Many of the companies which provide ECMO facilities stipulate that all the components of their system alone be utilized. Nonetheless, our experience vis-à-vis ECMO procedures with unmatched components from different companies, in consequence of economic sanctions imposed on our country, was successful at least in the short term. 

## Notes:

***This paper should be cited as: ***Ahmadi ZH, Salehi F, Niusha S, Raoufy MR, Farzanegan B, Afshar A, Mafhoomi Y, Faghih Abdollahi Z, Radmand G, Ansari Aval Z, Jahangirifard A. First Experience with Extracorporeal Membrane Oxygenation in Iran, under Difficult Conditions. J Teh Univ Heart Ctr 2018;13(4):166-172.

## References

[1] Bartlett RH (2014). John H Gibbon Jr Lecture. Extracorporeal life support: Gibbon fulfilled. J Am Coll Surg.

[2] Marasco SF, Lukas G, McDonald M, McMillan J, Ihle B (2008). Review of ECMO (extra corporeal membrane oxygenation) support in critically ill adult patients. Heart Lung Circ.

[3] Sharma AS, Weerwind PW, Maessen JG (2014). Extracorporeal membrane oxygenation resuscitation in adult patients with refractory septic shock. J Thorac Cardiovasc Surg.

[4] Jahangirifard A, Hossein Ahmadi Z, Golestani Eraghi M, Tabarsi P, Marjani M, Moniri A, Nadji SA, Hashemian SM, Velayati AA (2016). H1N1 influenza patient saved by extracorporeal membrane oxygenation: first report from Iran. J Teh Univ Heart Ctr.

[5] Ma P, Zhang Z, Song T, Yang Y, Meng G, Zhao J, Wang C, Gu K, Peng J, Jiang B, Qi Y, Yan R, Ma X (2014). Combining ECMO with IABP for the treatment of critically Ill adult heart failure patients. Heart Lung Circ.

[6] Patel JK, Schoenfeld E, Parnia S, Singer AJ, Edelman N (2016). Venoarterial extracorporeal membrane oxygenation in adults with cardiac arrest. J Intensive Care Med.

[7] MacLaren G, Combes A, Bartlett RH (2012). Contemporary extracorporeal membrane oxygenation for adult respiratory failure: life support in the new era. Intensive Care Med.

[8] Nguyen DQ, Kulick DM, Bolman RM, 3rd, Dunitz JM, Hertz MI, Park SJ (2000). Temporary ECMO support following lung and heart-lung transplantation. J Heart Lung Transplant.

[9] Ko WJ, Lin CY, Chen RJ, Wang SS, Lin FY, Chen YS (2002). Extracorporeal membrane oxygenation support for adult postcardiotomy cardiogenic shock. Ann Thorac Surg.

[10] Formica F, Avalli L, Martino A, Maggioni E, Muratore M, Ferro O, Pesenti A, Paolini G (2008). Extracorporeal membrane oxygenation with a poly-methylpentene oxygenator (Quadrox D). The experience of a single Italian centre in adult patients with refractory cardiogenic shock. ASAIO J.

[11] Beiras-Fernandez A, Deutsch MA, Kainzinger S, Kaczmarek I, Sodian R, Ueberfuhr P, Meiser B, Schmoeckel M, Reichart B, Brenner P (2011). Extracorporeal membrane oxygenation in 108 patients with low cardiac output – a single-center experience. Int J Artif Organs.

[12] Schopka S, Philipp A, Lunz D, Camboni D, Zacher R, Rupprecht L, Zimmermann M, Lubnow M, Keyser A, Arlt M, Schmid C, Hilker M (2013). Single-center experience with extracorporeal life support in 103 nonpostcardiotomy patients. Artif Organs.

[13] Park M, Azevedo LC, Mendes PV, Carvalho CR, Amato MB, Schettino GP, Tucci M, Maciel AT, Taniguchi LU, Barbosa EV, Nardi RO, Ignácio Mde N, Machtans CC, Neves WA, Hirota AS, Costa EL (2012). First-year experience of a Brazilian tertiary medical center in supporting severely ill patients using extracorporeal membrane oxygenation. Clinics (Sao Paulo).

[14] Hei F, Lou S, Li J, Yu K, Liu J, Feng Z, Zhao J, Hu S, Xu J, Chang Q, Liu Y, Wang X, Liu P, Long C (2011). Five-year results of 121 consecutive patients treated with extracorporeal membrane oxygenation at Fu Wai Hospital. Artif Organs.

[15] Zangrillo A, Landoni G, Biondi-Zoccai G, Greco M, Greco T, Frati G, Patroniti N, Antonelli M, Pesenti A, Pappalardo F (2013). A meta-analysis of complications and mortality of extracorporeal membrane oxygenation. Crit Care Resusc.

[16] Paden ML, Rycus PT, Thiagarajan RR, ELSO Registry (2014). Update and outcomes in extracorporeal life support. Semin Perinatol.

[17] Prodhan P, Gossett JM, Rycus PT, Gupta P (2015). Extracorporeal membrane oxygenation in children with heart disease and del22q11 syndrome: a review of the Extracorporeal Life Support Organization Registry. Perfusion.

[18] Aubron C, Cheng AC, Pilcher D, Leong T, Magrin G, Cooper DJ, Scheinkestel C, Pellegrino V (2013). Factors associated with outcomes of patients on extracorporeal membrane oxygenation support: a 5-year cohort study. Crit Care.

[19] Douglass BH, Keenan AL, Purohit DM (1996). Bacterial and fungal infection in neonates undergoing venoarterial extracorporeal membrane oxygenation: an analysis of the registry data of the extracorporeal life support organization. Artif Organs.

[20] O'Neill JM, Schutze GE, Heulitt MJ, Simpson PM, Taylor BJ (2001). Nosocomial infections during extracorporeal membrane oxygenation. Intensive Care Med.

[21] Burket JS, Bartlett RH, Vander Hyde K, Chenoweth CE (1999). Nosocomial infections in adult patients undergoing extracorporeal membrane oxygenation. Clin Infect Dis.

[22] Sun HY, Ko WJ, Tsai PR, Sun CC, Chang YY, Lee CW, Chen YC (2010). Infections occurring during extracorporeal membrane oxygenation use in adult patients. J Thorac Cardiovasc Surg.

